# An Evaluation of Methods for Inferring Boolean Networks from Time-Series Data

**DOI:** 10.1371/journal.pone.0066031

**Published:** 2013-06-21

**Authors:** Natalie Berestovsky, Luay Nakhleh

**Affiliations:** Department of Computer Science, Rice University, Houston, Texas, United States of America; University of Pittsburgh, United States of America

## Abstract

Regulatory networks play a central role in cellular behavior and decision making. Learning these regulatory networks is a major task in biology, and devising computational methods and mathematical models for this task is a major endeavor in bioinformatics. Boolean networks have been used extensively for modeling regulatory networks. In this model, the state of each gene can be either ‘on’ or ‘off’ and that next-state of a gene is updated, synchronously or asynchronously, according to a Boolean rule that is applied to the current-state of the entire system. Inferring a Boolean network from a set of experimental data entails two main steps: first, the experimental time-series data are discretized into Boolean trajectories, and then, a Boolean network is learned from these Boolean trajectories. In this paper, we consider three methods for data discretization, including a new one we propose, and three methods for learning Boolean networks, and study the performance of all possible nine combinations on four regulatory systems of varying dynamics complexities. We find that employing the right combination of methods for data discretization and network learning results in Boolean networks that capture the dynamics well and provide predictive power. Our findings are in contrast to a recent survey that placed Boolean networks on the low end of the “faithfulness to biological reality” and “ability to model dynamics” spectra. Further, contrary to the common argument in favor of Boolean networks, we find that a relatively large number of time points in the time-series data is required to learn good Boolean networks for certain data sets. Last but not least, while methods have been proposed for inferring Boolean networks, as discussed above, missing still are publicly available implementations thereof. Here, we make our implementation of the methods available publicly in open source at http://bioinfo.cs.rice.edu/.

## Introduction

The fate of a cell, and an organism as a whole, is determined by the functioning of a complex cellular machinery. Part of this machinery, referred to as the regulatory network, is comprised of molecular species (genes, proteins, micro RNA, etc.) and their interactions. This network, upon receipt of extracellular signals, relays signals from the cell membrane to the nucleus, and initiates a transcription process that controls the production and abundance of proteins. Proper functioning of these networks is essential to the survival and adaptation of all living organisms, while malfunctioning of these networks has been identified as the cause of various diseases. Therefore, elucidating these networks in different cells and organisms, and understanding their structural and dynamic properties under different conditions are major endeavors in biology. The sheer size and complexity of these networks make it essential to develop computational tools for automated inference, or learning, of these networks from experimental data.

Advanced biotechnologies have amassed large amounts of genomic and proteomic data to enable computational inference of parts of these regulatory networks. Various approaches for modeling and analyzing regulatory networks have been introduced, which differ in the levels of complexity they model and provide different advantages and limitations [1], [2]. These approaches vary in their assumptions and parameterization, and consequently expressiveness, from the very detailed (e.g., systems of ordinary differential equations) to the least detailed (e.g., graph connectivity). The tradeoff among criteria such as simplicity, scalability and expressiveness, highlights one of the central issues in systems biology, where defining the scope and abstraction level of the model highly depends on the availability of biological knowledge to incorporate into the model as well as the question of interest [3].

Boolean networks have emerged as a plausible model of regulatory networks (e.g., [4], [5]) that, on the one hand, does not require knowledge of the kinetic parameters, and on the other hand, provides important insight into the dynamics, as well as steady-state behavior, of the system. Their appeal lies in the fact that the Boolean relationships can be established from relatively small amounts of experimental data. Under a Boolean network model, the state of a gene is either ‘on’ or ‘off,’ and that state is updated according to a Boolean rule, or function, that relates the next-state of a gene to the current-state of the entire system. As such updates are executed for a number of steps, the dynamics of the Boolean network are simulated and Boolean trajectories of the model are produced. These trajectories can be compared to the experimental data for validation and refinement of the model. Further, the Boolean network can be manipulated to simulate *in silico* perturbations for generating testable hypotheses.

To account for stochasticity in gene regulatory systems [6], [7], probabilistic Boolean networks (PBNs) were introduced [8], [9]. Unlike deterministic Boolean networks, the next-state of a gene is determined by a Boolean function that is selected, with a certain probability, from a set of Boolean functions associated with that gene. While PBNs are more appropriate for modeling regulatory networks than deterministic Boolean networks, their application in practice has been very limited, mainly due to the complexity of computing the state transitions and steady-state distributions [10]. More broadly, a wide array of mathematical and computational techniques have been devised for modeling regulatory networks. These models differ, among other things, in the assumptions they make, the quality and quantity of experimental data they require, and in their expressive and predictive powers. For a recent survey of such classes of methods, the reader is referred to [1], [2]. In this paper, we focus on deterministic Boolean networks, and incorporate asynchronous simulations of these networks, which is aimed at capturing stochasticity.

The process of inferring the Boolean model from time-series data can be separated into two distinct steps. In the *first* step, the time-series data is discretized into maximally informative binary state transitions (which we refer to as “binarization”). The *second* step uses these binary profiles to learn the Boolean network that best captures the Boolean trajectories. Once a Boolean network is learned, it can be analyzed for structural and steady-state properties, simulated, in synchronous or asynchronous mode, to reveal the dynamics of the underlying system, or perturbed *in silico* to generate testable hypotheses.

In this paper, we evaluate the performance of methods that have been proposed for binarizing time-series data and for learning Boolean networks. We use the time-series data of four regulatory networks, three of which are synthetically generated and one consisting of true experimental data points. For the first step, we consider three pre-processing methods: (1) the 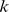-means clustering technique with 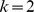, which was proposed in [11], (2) the BASC A method of [12], and (3) an *iterative 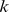-means* clustering method that we propose. For the second step of learning the Boolean network from binary data, we consider the three most commonly used methods: REVEAL [13], Best Fit [14], and Full Fit [15]. All these methods are reviewed briefly below. We assess the performance of combinations of these methods on four regulatory networks of varying sizes (in terms of the number of species and interactions) and dynamics complexity, report on the results, and make recommendations on their use. In particular, we find that our *iterative 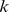-means* binarization method, combined with BESTFIT or REVEAL, produce best quality Boolean networks, with ability to capture even complex oscillatory dynamics. We also find that Boolean networks, when learned using the appropriate methods, have good predictive power. Our findings on the four systems disagree with the classification of [2], which puts Boolean networks on the lower end of the expressiveness scale. Last but not least, we make our implementation of the methods available publicly in open source at http://bioinfo.cs.rice.edu/. Our implementation allows the user to choose any combination of data binarization and network learning methods.

## Methods

In this paper, we are concerned with the problem of learning a Boolean network from time-series data. Let 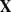 be an 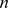-dimensional binary vector that represents the current state of the system. Each element 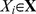 corresponds to the state (0 or 1) of species 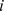. A Boolean network defined by a set 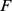 of 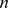 Boolean functions. For every 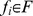, such that 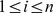, 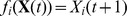. In other words, given a current state of the system 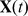, 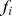 determines the (binary) value of species 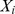 at time 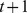. Given a Boolean network 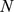 on 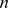 variables and an initial state 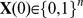, the dynamics of the system can be simulated by repeatedly applying the Boolean functions and updating the “current” state. In the classical synchronous simulation, the states of all variables are updated simultaneously *after all* of the functions in 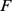 have executed, whereas in asynchronous simulation, the states are updated one at time by randomly choosing a function 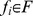 and updating the state of 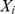 *immediately*. The final asynchronous simulation is the average across many executions. The latter technique belongs to the category of *single-molecule level models* [2]. This category of simulators is based on the stochastic simulation algorithm (SSA) [16], [17] which is widely accepted and frequently used. Roughly speaking, asynchronous simulation of a Boolean network amounts to executing SSA with a uniform distribution on the Boolean functions to be executed. Both synchronous and asynchronous simulation methods are used throughout this work.

The input of our problem consists of time-series data 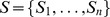 of 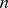 species, each of size 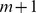, where 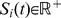 (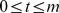) is the concentration of species 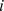 at time 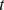, and the output is a Boolean network 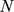 on the 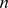 species (or a set, in the case that multiple optimal networks are found; we define optimality with respect to an error below). Approaches that learn 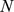 from 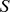 first binarize the time-series data, that is, turn 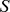 into binary trajectories 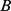 (one per species), and then learn the network from 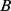. In this work, we evaluate the performance of two existing binarization techniques [11], [12] and an additional one that we devise, and evaluate three network learning methods [13]–[15] in all nine possible combinations, on four regulatory networks. The general outline of the learning approaches is given in Table 1 with detailed description of each step presented below. Since the two versions of 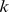-means are initialized randomly, multiple applications may result in different binarizations, and potentially different networks; hence the need for iterating 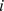 times in the algorithm (this iteration is neither needed nor performed when the binarization is done via the deterministic BASC A method).

### Binarization

#### Two clusters *k-means* binarization

(KM-1) directly clusters the time-series data into two clusters with all values in the cluster with the higher mean being set to 1, and the ones in the cluster with lower mean set to 0 [11]. This method is fast and effective for simple time-series; however, it could miss some of the features of the data especially in the presence of oscillations and fluctuations.

#### Iterative *k-means* binarization

**Figure 1:**
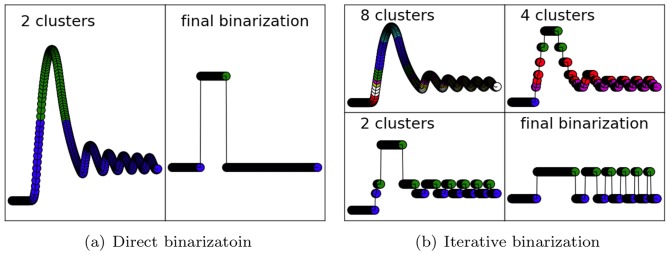
Iterative *k-means* clustering with 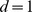 (direct binarization) vs. 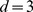. More refined binarization is achieved with higher values of 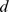.

(KM-3) is a new method we propose to address the shortcomings of of KM-1 when dealing with complex dynamics. In this method, we define a depth of clustering 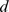, and set the initial number of cluster to 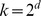. In each iteration, we classify the data into 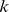 disjoint clusters 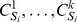; then, for each cluster 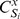, all its values are replaced by the cluster's mean 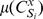. For the next iteration, we decrement the values of 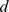 by one, and repeat the clustering on data from current iteration. This process continues until 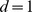, resulting in final binarization where the data in the cluster with higher values are replaced by 1, and the data in the the cluster with lower values are replaced by 0. In our analysis, we found that 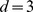 yields the best results for all systems we consider here. For 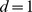, this method is equivalent to KM-1. Fig. 1 illustrates the advantages of iterative *k-means* clustering, while halving the value of 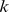 in each iteration, over direct use of *k-means* to acquire two clusters. Direct clustering into 2 clusters misses the oscillations in the data, whereas the iterative application of 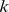-means successfully captures it.

**Table 1:** Algorithm 1 From Time-series to Boolean Networks.

**Input:**
• Time-series 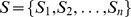 of 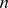 species;
• Binarization method 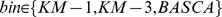 ;
• Learning method 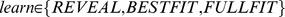 ;
• Error scoring metric 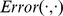 ;
• Number of iterations 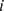 ;
**Output:**
All Boolean networks that are optimal under the error metric;
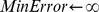 .
**Repeat** 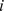 times:
1. 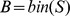 ;
2. Remove redundancy in 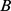 ;
3. 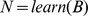 ;
4. **If** 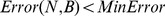
(a) 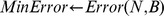 ;
Return all Boolean networks 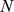 with 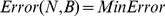 .

#### BASC A binarization

(BASC A) first converts the vector of time-series measurements into an ordered, ascending step function 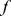 of size 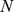 with 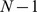 discontinuities. It, then, uses dynamic programming to calculate optimal step functions with 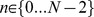 discontinuities by minimizing the Euclidean distance to the initial step function 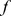. Further, in each step function, the algorithm finds the strongest discontinuity 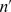 using the scoring metric that favors large jump size (characterized by the difference between the average of all 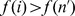 and average of all 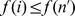) and the lower approximation error with respect to the original 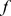 (the sum of the quadratic distances of all data points using 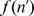 to determine the potential threshold). When the vector 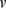 of all strongest discontinuities has been identified, BASC A determines the final threshold by using the median value in 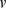. For full details, the reader is referred to [12].

In both *k-means* binarization methods, the initial cluster centroids are chosen at random. Multiple runs of these binarization methods can lead to different binary profiles 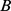 and, potentially, to different Boolean networks 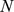. BASC A, on the other hand, is deterministic. Different runs with BASC A may still result in different networks due to sampling in the Boolean network learning algorithms.

### Redundancy removal

The *steady state* of a Boolean network is obtained when we have two equal consecutive states; that is, 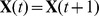. However, in practice, it may be that data is measured at a very fine time-scale, giving a false indication of steady-state signal, especially when abstracted into binary values. Therefore, it is important that the binarized data is processed so as to remove “false steady-state” transitions, while maintaining the true steady-state transition. Since a steady-state is a point attractor, a pair of equal consecutive states is indicative of a true steady state only if it was the last pair in the series. Therefore, except for the last pair in the series, we remove from each maximal consecutive sequence of identical states all but one of the states. We also considered a reduction techniques proposed by [18], where the authors first determine the average number of bits needed to consider a transition *significant* and then reduce the binary profiles to only keep the *significant* transition changes. This method is equivalent to our approach if only 1 bit is needed to mark a *significant* transition. However, we found that when the average number of bits needed is above 1, this reduction method skips some of the informative transitions that could be used in the Boolean network learning step.

### Learning a Boolean network

#### REVEAL

(REVEAL) [13] uses deterministic transition table to infer the Boolean relationships between the variables. First, additional data pre-processing of converting binarized profiles 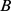 into transition table is needed. It is possible that in 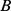 we may have pairs of transitions 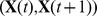 and 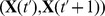, where 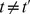, 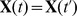, and 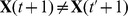. This scenario amounts to nondeterministic transition tables, and cannot be handled by REVEAL. To address this, if there are transitions from state 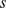 leading to 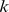 possible states 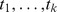, we count the number of times each transition 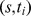, 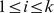, is observed, keep the pair 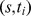 with the highest count, and remove the rest. This eliminates nondeterminism from the transition table. Using the resulting transition table, for each variable 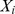, REVEAL computes the entropy value 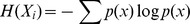, where 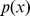 is the probability of observing value 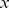 (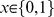) for variable 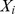 [19]. Further, for each subset 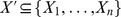 of variables, REVEAL computes the mutual information between 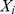 and 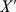 as
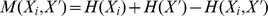
where 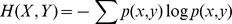 is the joint entropy of 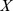 and 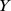. The smallest (in terms of size) subset 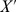 that yields 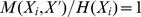 reflect the set of genes whose states determine the next state of the gene represented by variable 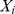. To resolve, the function 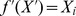 is assigned only in the case if it is complete (all permutation of 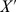 are represented), and discarded otherwise. REVEAL algorithm works incrementally by first checking how well each single gene determines the value of 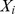 (for every 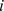), then checks every pair of genes, then every triplet of genes, and so on. In [13], the authors recommended not considering subsets 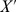 with more than three genes. This maximum input size was shown in original work of Liang *et al.* to be most effective in terms of both biological plausibility and inability to be further reduced.

#### Best-Fit Extension

(BESTFIT) determines, for each Boolean variable 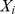, the set 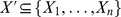 of size 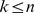, that best explains 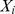 with the least *error size*. The algorithm utilizes *partially defined Boolean function* pdBf(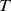,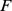), where 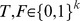, and denote the set of true and false examples, as extracted from binarized time-series data. For each time step 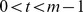, unique occurrences of pairs 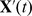 and 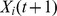 are added to pdBf(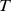,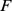), such that 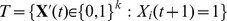 and 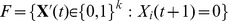. Further, the *error size* 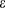 is defined by the number of inconsistencies within pdBf(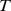,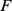) and is determined by size of the intersection of the sets 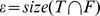. The 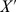 with the lowest error is chosen and the undefined entries in the corresponding pdBf(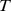,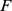) are randomly assigned to extract a deterministic function. For full details, the reader is referred to [14]. Similar to REVEAL, BESTFIT also works incrementally, resolving 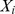 with 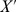 of smallest size and discarding the rest.

#### Enumeration inference method

(FULLFIT) determines the set 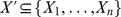 that fully explains the variable 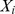. Similar to BESTFIT, for each time step 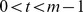, unique occurrences of pairs 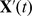 and 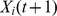 are added to pdBf(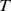,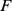), and the number of inconsistencies 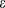 is calculated. The only difference here is that algorithm only accepts the functions with 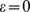 [15]. Ideally, after all possible, fully consistent, functions are gathered, all resulting networks can be enumerated by choosing a single function for each 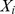. However, in practice it could quickly becomes computationally infeasible. To address that, Martin *et al.* suggest sampling networks from the pool of functions [15].

In all three methods it is often the case that a single 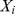 can have multiple 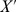 of the same size that determine it. This happens because the original data is incomplete, as the binarized time-series might not have *all* possible input-output pairs represented in it. The incomplete transition table allows all algorithm to match each 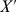 to multiple functions 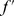. Therefore, for a given binarization, we sample 100 networks and assess them to find the one with *minimum error for a single binarization*.

### Error assessment

As a Boolean network is learned by one of the three algorithms, we need to assess its quality, or fit for the data. Let 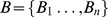 be a set of binary trajectories of equal size used to infer a Boolean network 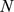. We execute 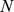 using a synchronous Boolean simulator [20] to generate a binary trajectory 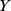 whose length is equal to that of 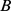, and whose first state is identical to that given by 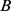. In a synchronous simulation, all states are updated simultaneously after execution of all the Boolean functions. The use of this simulator further illustrates the need to remove redundancy in the binary data, as the system cannot stay in the same state unless for the steady state. We then define the error of Boolean network 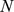 with respect to data 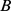 as
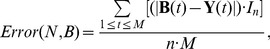
(1)where 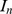 is an 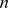-dimensional vector of all ones, and 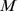 is the number of states in the binarized, reduced time series. A network with smaller error better captures the system. Zero error can be achieved for some, but not all, networks, indicating an inferred Boolean network that perfectly matches the reduced binarized data.

## Results

In this section, we evaluate the performance of each *bin:learn* combination in Table 1 on four time-series data sets, one of which is experimental and the other three are synthetically generated from regulatory network kinetic models. The data sets vary in the number of species, the number of data points, as well as in the complexity of the dynamics; we discuss the implications of size and complexity in the next section.

The first system we analyzed consists of a regulatory network of four genes, adopted from [21]. In this network, gene 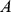 is self-regulatory, the protein products of genes 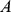 and 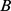 form a heterodimer that activates the expression of gene 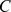, the protein product of gene 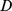 activates the translation of the protein product of 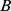, and the protein product of 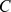 inhibits the expression of genes 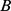 and 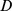. The system of ordinary differential equations (ODEs) that we used to model the toy network from [21] is:
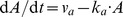


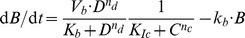


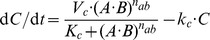


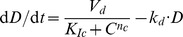



**Figure 2:**
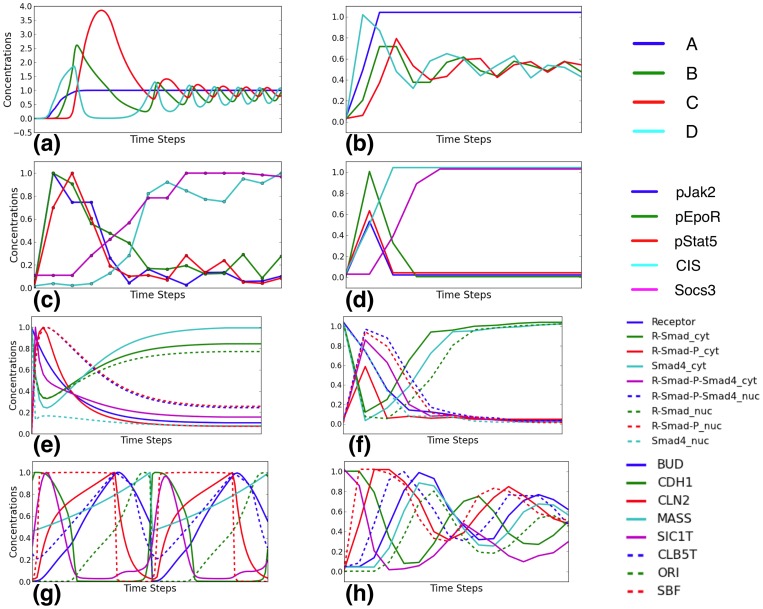
True dynamics (left column) and the dynamics based on asynchronous simulation of the best-scoring Boolean networks learned from the data (right column) of the four systems: toy network (a–b), Jak-Stat (c–d), Smad (e–f), and budding yeast cell cycle (g–h). The Boolean network simulated for each system is one with minimum error obtained by the KM3:REVEAL method (see Table 2).

To generate the time-series data shown in Fig. 2(a) for this system, we used the following values for the parameters: 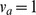, 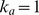, 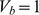, 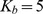, 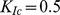, 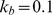, 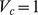, 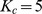, 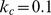, 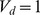, 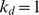, 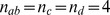. Further, we used an initial condition of 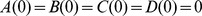. We solved the ODEs numerically by using the *ode45* built-in function in Matlab. This network exhibits complex, oscillatory dynamic behaviors, as can be shown in Fig. 2(a).

For the second system, we used an experimentally derived time-series data set for a *Jak2/Stat5* signaling network with negative regulatory feedback loops. The dynamics of this pathway were explored by Bachmann *et al.* to determine the roles of the two transcriptional negative feedback regulators *Socs3* and *CIS* [22]. For this system, we obtained the time-series data from [22], which are shown in Fig. 2(c). The experimental data shows a spike in the activity of the phosphorylated signaling components (*pJak2*, *pEpoR*, and *pStat5*) at the initial time points, but later suppression by the inhibitory feedback from the expressed genes *Socs3* and *CIS*.

For the third system, we used an *Smad* network [23]. *Smad* proteins are an important intracellular mediator of 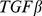 signaling, a system that plays a significant role in cell growth and differentiation. Upon 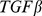 stimulation, *Smad* proteins accumulate in the nucleus and regulate the transcription of target genes [23]. This system is relatively much larger than the two systems we have analyzed thus far. To produce time-series data for the *Smad* system, we acquired a curated COPASI model from the *Biomodels* database [24]. Using this model, we obtained time-series data in Fig. 2(e).

The fourth system we analyzed is one similar to the *Smad* system in terms of the number of species, yet more complex in terms of dynamics. The budding yeast cell cycle is a classic example of the sequence of events during which a growing cell replicates. The system has been mostly worked out in a consensus set of interactions. Chen *et al.* developed a system of ODEs to model this consensus hypothesis [25]. The synthetic data for the *Smad* and cell cycle systems were generated using COPASI [26]. The dynamics of the budding yeast cell cycle are shown in Fig. 2(g). The original time series is normalized between 0 and 1.

### Ability to model dynamics: Expressiveness of the learned Boolean networks

For each *bin:lean* combination in Table 1, we execute 100 searches of minimum-error Boolean networks, according to Eq. (1), where each search is run for up to 10,000 iterations (the search is terminated whenever a minimum error of 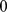 is achieved, since this is the best possible value). For each of the four systems, we report Min error, Convergence, Uniqueness, and Correctness, in Table 2. Min error is the minimum error achieved by a Boolean network over the 100 searches. For Convergence, the value indicates the number of iterations it took each of the 100 searches to identify the minimum-error Boolean network, averaged by the number of searches (100). Hence, a value of 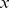 for Convergence indicates that, on average, it took each search 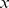 iterations to learn the minimum-error Boolean network. The lower this value, the better the performance of the method. The Uniqueness value indicates the number of distinct Boolean trajectories produced, or captured, by the learned Boolean networks. The upper bound on this value is 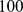, and that would indicate that all learned Boolean networks have distinct trajectories. A value of 1 indicates that all Boolean networks learned capture exactly the same dynamics (this does not necessarily mean that they are identical Boolean networks, though). In addition to systematically computing the error between the “true dynamics” and the dynamics of the learned Boolean networks, using Eq. (1), we also visually inspected the dynamics and checked if they match (mainly by comparing the curves); this is the Correctness entry in Table 2, which takes value ‘ Y’ if the true dynamics and the asynchronously simulated dynamics of the learned Boolean network match, and ‘ N’ otherwise. The reason we conducted a visual inspection is because automatically comparing two time series is not a trivial task, and even existing measures suffer from exaggerating the difference between two time series in some cases, and diminishing that difference in other cases (e.g., imagine comparing two time series data sets whose only difference is that all times points in one are shifted by some constant).

**Table 2:** Evaluation results for different combinations of binarization and learning methods on the four networks.

		Toy network			Jak-Stat			Smad			Cell cycle		
		KM-1	KM-3	BASC A	KM-1	KM-3	BASC A	KM-1	KM-3	BASC A	KM-1	KM-3	BASC A
REVEAL	**M**in error	0.43	0.007	0.025	0.28	0.0	0.26	0.48	0.0	0.73	0.52	0.012	0.52
	**C**onvergence	1	14	1	595	2237	2	1	12	1	6	559	1
	**U**niqueness	1	1	1	1	3	1	1	96	1	1	6	1
	**C**orrectness	N	Y	N	N	Y	N	N	Y	N	N	Y	N
BESTFIT	**M**in error	0.13	0.007	0.125	0.0	0.0	0.26	0.48	0.0	0.73	0.05	0.005	0.05
	**C**onvergence	1	17	1	85	1	1	1	1	1	6	8	1
	**U**niqueness	1	1	1	1	6	1	23	96	1	1	53	1
	**C**orrectness	N	Y	N	Y	Y	N	N	Y	N	Y	Y	Y
FULLFIT	**M**in error	0.43	0.15	0.7	0.0	0.0	–	0.48	0.0	0.73	0.4	0.08	0.4
	**C**onvergence	1	18	1	104	1	–	1	1	1	6	741	1
	**U**niqueness	1	1	1	1	6	–	23	96	1	1	6	1
	**C**orrectness	N	N	N	Y	Y	–	N	Y	N	N	N	N

Table 2 shows the performance of the nine *bin:learn* combinations of Table 1 on the four systems. For the *Jak-Stat* and *Smad* systems, a minimum error of 0 was achievable, whereas for the toy network the lowest possible error achievable was 0.007 and for the cell cycle system it was 0.005. As the Correctness values indicate, the minimum-error Boolean networks always produced dynamics similar to the true dynamics, as revealed by visual inspection. The exception to this trend are the Boolean networks learned using the BASC A binarization on the toy network, in which case minimum-error networks were learned, but their dynamics trajectories looked different from the true ones. Further, for the budding yeast cell cycle system, learned Boolean networks with error up to twice the minimum error achievable produced similar dynamics to the true ones. More generally, the combination KM3:BESTFIT always generated the Boolean network with the minimum possible error, followed by KM3:REVEAL. The repetitive nature of KM3 makes it perform well for systems with oscillatory dynamics. The dynamics produced from the best-scoring learned network on each of the four systems are shows in the right column of Fig. 2.

For the *Jak-Stat* system, the BASC A:FULLFIT combination did not produce a single network for this case. This could only happen if every considered function 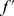 contained non-determinism. On the other hand, KM3 is able to achieve zero error with all learning methods. For the *Smad* system, binarizing the data with KM3 resulted in learning zero-error networks, regardless of the learning method. For the cell cycle system, the BESTFIT learning method resulted in minimum-error networks, regardless of the binarization method.

In terms of convergence, both the binarization and the learning method have an effect. For example, in the case of the *Jak-Stat* system, using KM3:BESTFIT converges in 1 iteration, whereas using KM3:REVEAL requires on average 2,237 iterations. In this case, the learning method makes a big difference. However, it is important to note that if we take the minimum error and correctness into account (that is, require that the learned network has the minimum error and that its dynamics match the true ones by visual inspection), then KM3 is the best, in terms of convergence, across all systems and learning methods, except in the case of the cell cycle system, where KM1 results in a faster convergence than KM3, when combined with BESTFIT for learning; however, the difference is only 2 iterations, which is negligible.

In terms of uniqueness, the only system on which multiple distinct Boolean networks were learned is the *Smad* system. What characterizes this system is that it is large in terms of the number species, yet has very simple dynamics. This is analogous to the “too many variables, too few equations” case in solving systems of equations, where the degree of freedom is very large, and non-uniqueness of solutions naturally arises.

Dynamics of the learned Boolean networks (using the KM3:REVEAL combination), as obtained by asynchronous Boolean simulation using the tool of [20], match the true dynamics very well, for the most part, as shown in Fig. 2.

### Faithfulness to biological reality: The learned Boolean functions

The binarization and redundancy removal steps described above result in the removal of data, and with this removal comes loss of information. This loss of information undoubtedly affects, to varying degrees, the match between the learned Boolean network itself and the rules governing the true system underlying the time-series data. Further, the fact that the number of Boolean functions is exponential in the number of genes in the system combined with the fact that in practice very few data points are available to learn these networks give rise to a situation where multiple Boolean networks with equal score (or, error) can be learned. Indeed, in [15] for example, the authors discussed the issue that multiple “optimal” networks were learned and that a summary of these networks could be presented. However, two important points are worth mentioning here. First, in practice, the regulatory network is unknown, and judging whether a Boolean network provides a close approximation or not is not easy to do objectively. Second, in our study, and other similar studies of modeling techniques, ordinary differential equations are used as a proxy of the real system and are used to simulate the “true dynamics.” When comparing a learned Boolean network, or any other model, to these ODEs, it is important to keep in mind that these ODEs themselves are not necessarily unique with respect to the dynamics they generate (that is, many other systems of ODEs, some simpler and some more complex, could generate the same dynamics). Thus, assessing the faithfulness of learned Boolean networks to biological reality must be done with these two points in mind.

In addition to the dynamics of the learned networks, we also inspected the Boolean functions that the methods learned for each of the systems. We discuss here only the networks learned for the toy network and *Jak-Stat* system. For the toy network, the unique zero-error Boolean network obtained by KM3:REVEAL consists of the functions: 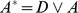, 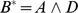, 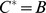, 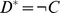 (where 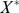 denotes the *next* state of variable 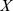). These Boolean rules capture many of the assumptions of the network (e.g., 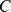 inhibits 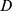, 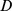 activates 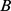). While there is no direct involvement of 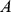 in the regulation of 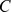, its effect is captured indirectly via 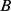, which has 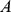 in its regulatory function. Further, in the Boolean function for updating 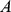, the network captures the fact that 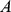 is self-regulatory. Indeed, if 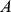 is treated as an input to the system and set to 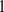, the Boolean functions learned are: 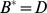, 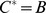, 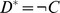. Keeping the two points we raised above in mind, the question is, for example: Since the Boolean rule 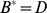 conveys no effect of 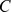 on 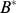, how would the “true dynamics” change if we remove the 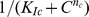 term from the equation for 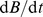
? We performed this test and the results showed that the change to the “true dynamics” was negligible (too negligible to make a difference in the Boolean network learned from the data!).

For the *Jak-Stat* system, KM3:REVEAL produces three Boolean networks that differ only in the function controlling 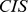. The functions learned by this method are:
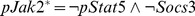


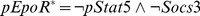


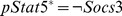


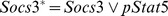


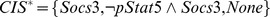
While this Boolean network is much simpler than the detailed model constructed in [22], it does capture several of the reactions highlighted in that model. For example, in [22], the authors assumed that 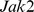 is inhibited by 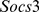, which is captured in the inferred Boolean network. For the other reactions, the Boolean network inferred “short-circuited” versions (that is, indirect effects are inferred as direct ones). This Boolean network further reveals that 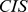 does not play a role in regulating the other molecules and, rather, 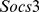 is the more central regulator.

It is important to note here that the original model of [22] was very detailed and incorporated much information from the literature. In our case, the model is learned simply from one time-series data set (Fig. 3 in [22]) without any additional knowledge to guide the inference of the Boolean functions. It is very important to note here that the parameters of the model (e.g., rate constants and initial concentrations) can have a significant effect on the the topology and functions of the inferred Boolean network. To illustrate, consider a biological network with a reaction in which 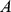 has an inhibitory effect on 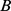, which would be inferred as a Boolean rule of the form 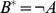. If the concentration of 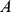 in the experiment or the reaction rate is too low, the experimental data might not exhibit the actual effect of 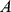 on 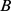, resulting in an inferred Boolean network that might neither capture 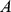 as an effector of 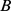, nor that the effect is inhibitory.

**Figure 3:**
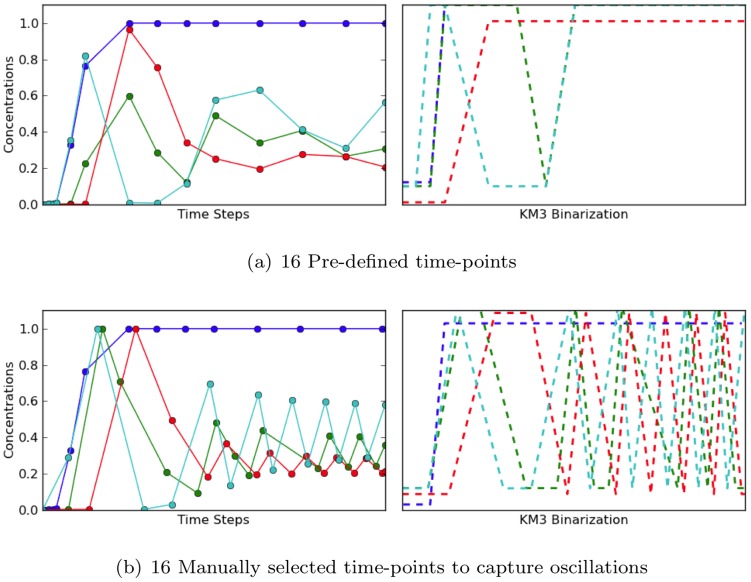
Dynamics of Boolean networks learned from 16 time-points of the toy network. (a) Time points correspond to 0 min, 5 min, 15 min, 30 min, 45 min, 1 hr, 2hr, 3hr, 6hr, 8 hr, 10 hr, 12 hr, 15 hr, 18 hr, 21 hr, 24 hr. (b) Time points are manually selected to capture the oscillatory patterns of the original system. Left panels show the time points selected, and right panels show the binary data obtained by applying KM3 to the measurements at the selected time points in the left panels. Binarized data are shifted vertically for readability. Blue, green, red, and cyan curves correspond to species A, B, C, and D, respectively.

### The amount of data needed for modeling

In [2], Boolean networks were placed on the lower end of the spectrum in terms of the amount of data needed for modeling (in our context, the amount of data is the number of time points at which molecular concentrations are measured in an experiment). However, even for a very abstract model of regulatory networks, such as Boolean networks, we hypothesize that the amount of data required to capture the dynamics is a function of the complexity of those dynamics in the underlying (unknown) system. Of the four systems we considered here, two exhibit simple dynamics (the *Jak-Stat* and *Smad* systems) and two exhibit complex, oscillatory dynamics (the toy network and the cell cycle system).

As we have seen already, all KM3:* combinations were successful at capturing the dynamics of the *Jak-Stat* system from a relatively small experimental data set with 14 time points. To explore the effect of the samples data points on the learned networks, we reanalyzed the toy network in two different ways. First, we generated 8- and 16-time-point data sets by dividing the time-series data to reflect measurements at 0 min, 5 min, 15 min, 30 min, 45 min, 1 hr, 2 hr, 3 hr, 6 hr, 8 hr, 10 hr, 12 hr, 15 hr, 18 hr, 21 hr, and 24 hr. Second, we manually selected time points to capture the oscillatory patterns of the original system (this is not doable in real data analyses, but we do it here to see if “optimal” choice of data points would result in good networks).

We observed similar results to those of the *Jak-Stat* system on the *Smad* system, where a few data points were sufficient to learn Boolean networks that capture the true dynamics. However, the situation is very different for the other two systems that exhibit more complex dynamics.

Figure 3 shows the dynamic trajectories for the toy network as interpolated from the sampled data points (left panels), as well as the binary version of these dynamics as obtained by KM3 (right panels).

As Figure 3(a) shows, the points selected from pre-defined time steps do not represent the dynamics of the oscillations and the resulting binary data is of very poor quality. Figure 3(b) shows the points that were manually selected to capture the peaks and troughs of the oscillation. However, even with manually selected points, none of the three methods (with KM3 binarization) were able to produce this oscillatory patterns in the Boolean network. This illustrates that a large number of time points is needed to learn a good Boolean network when the dynamics are complex. For example, we found that for the budding yeast cell cycle system at least 50 data points are needed to accurately capture the dynamics. But it is important to keep in mind that this system is heavily oscillatory.

### Predictive power of the learned Boolean networks

To validate the predictive power of Boolean networks, we conducted 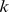-fold cross-validation experiments for the toy network, as well as the *Smad* and cell cycle systems. For each of these three systems, we used the mathematical models to generate 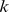 data sets, each differing in the initial state, used 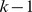 of these data sets to learn the Boolean networks, and used the remaining single data set for validation. That is, we treated each of 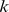 data sets here as a subsample. This procedure was repeated more than 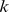 times for each system (to account for the potential non-uniqueness of the solutions): 100 times for the toy network, and 50 times for both the *Smad* and cell cycle systems.

For the toy network, we used the set of ODEs to generate 16 different data sets, corresponding to every possibility of setting the initial concentrations of the four species in the system to 0 or 1. For the *Smad* system, we used the COPASI model to produce six additional data sets that differed in the initial concentration of *RSmad-cyt*, *RSmad-nuc*, *Smad4-cyt*, and *Smad4-nuc*. The initial states of the 7 data sets are: (1,1,1,1), (0,1,1,1), (1,0,1,1), (1,1,0,1), (1,1,1,0), (0,0,1,1), and (1,1,0,0), where the four entries in each tuple correspond to the four molecules, respectively. For the budding yeast cell cycle system, we identified four “input” species (MASS, ORI, CLN2, and CDH1) and generated four additional data sets, using the COPASI model, that differed in the initial states of these species, for a total of five data sets with initial states: (0,0,0,1), (1,0,0,1), (0,1,0,1), (0,0,1,1), and (0,0,0,0).

The validation step was conducted as follows. Let 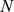 be a Boolean network learned from 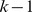 data sets, and let 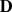 be the 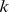-th time-series data set. First 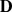 is binarized to generate the binary trajectories; call this 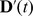, and assume 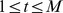. Then, for each pair of consecutive states in 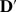, that is 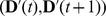, we synchronously execute the Boolean rules of 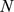 on state 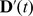 for one step, thus obtaining a new binary state 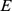, compare 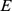 to 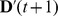, and compute the fraction of the number of entries in 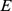 that are different from 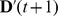. This is repeated for every 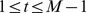, and the results are summed and divided by 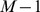. This procedure results in error values between 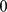, indicating the Boolean network is a perfect predictor, and 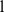, indicating the Boolean network makes wrong predictions all the time.

For Boolean network inference, we used the KM3:REVEAL and KM3:BESTFIT combinations, as these produced the best results in other experiments, as discussed above. For the toy network, the two methods produced Boolean networks with prediction error of 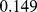 and 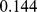, respectively. For the *Smad* system, the two methods produced Boolean networks with prediction error of 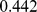 and 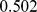, respectively. For the cell cycle system, the two methods produced Boolean networks with prediction error of 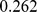 and 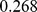, respectively.

Clearly, Boolean networks have very good predictive power of about 86% on the toy network, and a good predictive power of about 74% on the cell cycle system. The predictive power on the *Smad* system is poorer, reaching only about 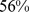 in the case of KM3:REVEAL and about 50% in the case of KM3:BESTFIT. To understand this poor predictive power, we investigated the seven data sets we generated for the cross validation experiment, and found that the times series across these data sets had a much larger variability than those for the other two systems.

### Conclusions

In this paper we studied the performance of methods for inferring Boolean networks from time-series data. Separating this problem into two steps, binarization and learning, we introduced a new method for binarizing time-series data, and evaluated the performance of methods within a single framework. We demonstrated the effectiveness of each method combination by analyzing four data sets that vary in size and dynamics complexity. We further demonstrated that proper binarization is crucial for the learning method to produce the correct network. This is demonstrated by the varying degree of success of the FULLFIT learning method, as it sensitive to any non-determinism that may result during binarization. We also observe that the randomized binarization obtained by 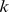-means clustering, especially KM3, results in better Boolean networks than ones learned from data that is binarized using BASC A. Most importantly, we found that the two combinations that performs best across all systems are KM3:REVEAL and KM3:BESTFIT. While the latter is much faster in terms of convergence, both are capable of capturing time-series trends very well. Fast convergence of BESTFIT can be explained by its lack of requirement that the candidate functions 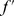 be complete. REVEAL, on the other hand, only accepts complete functions; consequently, it produces more intuitive Boolean networks, yet at the cost of time to converge. Our results show that, when learned properly from time-series data, Boolean networks can capture the dynamics to a high degree of accuracy, and can provide good predictive power. Further, depending on the complexity of the dynamics in the underlying network to be learned, the amount of time points at which concentrations must be sampled may be very large (which disagrees with the commonly stated claim that Boolean networks require very little data to learn or train).
